# Van Der Waals Semiconductor Based Omnidirectional Bifacial Transparent Photovoltaic for Visual‐Speech Photocommunication

**DOI:** 10.1002/advs.202306408

**Published:** 2023-12-11

**Authors:** Naveen Kumar, Thanh Tai Nguyen, Junsik Lee, Malkeshkumar Patel, Priyanka Bhatnagar, Kibum Lee, Joondong Kim

**Affiliations:** ^1^ Photoelectric and Energy Device Application Lab (PEDAL) and Multidisciplinary Core Institute for Future Energies (MCIFE) Department of Electrical Engineering Incheon National University Incheon 22012 Republic of Korea; ^2^ Solarlight Ltd. 119 Academy Rd. Yeonsu Incheon 22012 Republic of Korea

**Keywords:** field of view, omnidirectional, photocommunication, transparent photovoltaics, van der Waals

## Abstract

Omnidirectional photosensing is crucial in optoelectronic devices, enabling a wide field of view (wFoV) and leveraging potential applications for the Internet of Things in sensors, light fidelity, and photocommunication. The wFoV helps overcome the limitations of line‐of‐sight communication, and transparent photodetection becomes highly desirable as it enables the capture of optical information from various angles. Therefore, developing a photoelectric device with a 360° wFoV, ultra sensitivity to photons, power generation, and transparency is of utmost importance. This study utilizes a heterojunction of van der Waals SnS with Ga_2_O_3_ to fabricate a transparent photovoltaic (TPV) device showing a 360° wFoV with bifacial onsite power production. SnS/Ga_2_O_3_ heterojunction preparation consists of magnetron sputtering and is free from nanopatterning/nanostructuring to achieve the desired wFoV window device. The device exhibits a high average visible transmittance of 56%, generates identical power from bifacial illumination, and broadband fast photoresponse. Careful analysis of the device shows an ultra‐sensitive photoinduced defect‐modulated heterojunction and photocapacitance, revealed by the impedance spectroscopy, suggesting photon‐flux driven charge diffusion. Leveraging the wFoV operation, the TPV embedded visual and speech photocommunication prototype demonstrated, aiming to help visually and auditory impaired individuals, promising an environmental‐friendly sustainable future.

## Graphical abstract

A heterojunction of van der Waals (vdW) SnS with Ga_2_O_3_ transparent photovoltaic (TPV) device shows a 360^o^ wFoV with bifacial onsite power production and its real‐time application in visual‐speech photocommunication aiming to help visually and auditory impaired individuals, promising an environmental‐friendly sustainable future. The device shows an ultra‐sensitive photoinduced defect‐modulated heterojunction and photo capacitance, suggesting photon‐flux‐driven charge diffusion

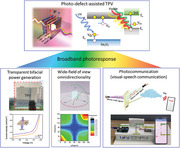

## 1 Introduction

Van der Waals (vdW) materials have gained remarkable recognition due to their unparalleled advantages such as robust light‐matter interaction,^[^ ^1^, ^2^ ^]^ desirable bandgap,^[^ ^3^, ^4^ ^]^ surface free from dangling bonds,^[^ ^5^ ^]^ high carrier mobility,^[^ ^6^ ^]^ exceptional flexibility for bending,^[^ ^7^ ^]^ and seamless integration capabilities.^[^ ^8^ ^]^ Consequently, these materials have emerged as the most promising candidates for constructing future optoelectronic devices, surpassing the capabilities of conventional 3D materials. Among different vdW materials, SnS has gained significant interest. Extensive research has been conducted on the SnS vdW semiconductor for energy and photodetection applications.^[^ ^9^, ^10^, ^11^ ^]^ While wireless photocommunication (PC) plays a pivotal role in information and technology, exploring vdW semiconductors in this area remains unexplored.^[^ ^12^, ^13^ ^]^ Investigating their potential for efficient wireless PCs is crucial, particularly in achieving omnidirectional /wide field of view (wFoV) photodetection. Owing to the high surface‐to‐volume ratio and its intrinsic nanostructure, SnS can be of great importance in highly photosensitive omnidirectional photodetection.^[^ ^14^ ^]^ Omnidirectional photosensing with a wFoV offers significant advantages by enabling the reception of photo signals from a broad angular range, surpassing conventional photodetectors’ limitations, and providing freedom from line‐of‐sight communication architecture.^[^ ^15^, ^16^, ^17^ ^]^ The wFoV can assist in various applications that can be used to create hypothetical 3D visual worlds. However, achieving 360° field of view (FoV) with a single photodetector or sensor has remained a significant challenge.^[^ ^18^ ^]^ Light Detection and Ranging (LIDAR), a versatile detecting and ranging system, comprises 64 lasers containing photodetectors. A pair of lasers and a detector are aligned to provide a vertical FoV of 28.6^o^. Consequently, the entire unit is rotated at a speed of up to 900 rotations per minut to obtain 360° FoV.^[^ ^19^ ^]^

In recent years, several heterojunctions and approaches have been employed to obtain omnidirectional photodetection. Photon management owing to the incorporation of nanostructures on top of electronic devices has shown an improved angular response.^[^ ^20^, ^21^ ^]^ For instance, Dong et al.^[^ ^22^ ^]^ fabricated a complicated and multi‐step omnidirectional photodetector. The structure was started from Zn wire to the formation of ZnO nanowire by hydrothermal followed by dipping in perovskite, PEDOT, carbon nanotubes, and PDMS, obtaining a fiber‐shaped ultraviolet (UV) photodetector. Fiber‐based photodetector demonstrated the dark‐to‐photocurrent ratio of two with the photocurrent generation of 2 nA under illumination. Kataria et al.^[^ ^23^ ^]^ developed a visible blind near‐infrared photodetector based on lanthanide‐doped upconversion nanoparticles on micro pyramidal PDMS. The device exhibited high photoresponsive characteristics to NIR exhibiting angular photoresponse from 90 to ±20°. Similarly, Lien and coworkers developed a ZnO nanoshell sphere for omnidirectional photodetection.^[^ ^24^ ^]^ Owing to its shape, the device showed photodetection to the angle of incidence from 0 to ±60°. All these reports were on developing an omnidirectional photodetector that shows photoresponse either UV or near‐infrared (NIR) with limited FoV but lacks power generation, which is the prime challenge to tackle with the growing need for high energy consumption.^[^ ^25^, ^26^ ^]^ Hence, a suitable choice of junction that provides features such as onsite energy production, omnidirectional broadband photodetection, and high transparency is essential for future optoelectronic devices and communication systems.

Here, using the novel and unique properties of vdW semiconductors, such as temperature‐sensitive layer orientation formation and high surface‐to‐volume ratio, omnidirectionality can be achieved without relying on nanostructuring or nanopatterning of the material/device.^[^ ^27^, ^28^ ^]^ Moreover, choosing suitable counter n‐type semiconductors may provide the advantages of onsite energy production. Here, Ga_2_O_3,_ a wide bandgap intrinsically n‐type material, can be suitable to intrinsic p‐SnS.^[^ ^29^, ^30^ ^]^

This study presents an omnidirectional vdW semiconductor‐based transparent photovoltaic (TPV) that exhibits bifacial power production with 360° FoV. Unlike conventional photovoltaic devices, the TPV presents high optical transparency and power production, an essential feature for self‐powered wireless photocommunication. The proposed TPV does not require complicated fabrication processes, such as nanostructure fabrication, nanostructure patterning, or various optical elements, to obtain omnidirectionality. The proposed TPV generates electrical power of 0.4 mW cm^−2,^ sufficient to power various IoT platforms. A real‐time application for visual and speech communication for visually and auditory impaired patients has been developed to receive signals from all directions and decipher them into visual text and sound.

## 2 Results and Discussion

### 2.1 Material Characterization and Analysis

**Figure 1:**
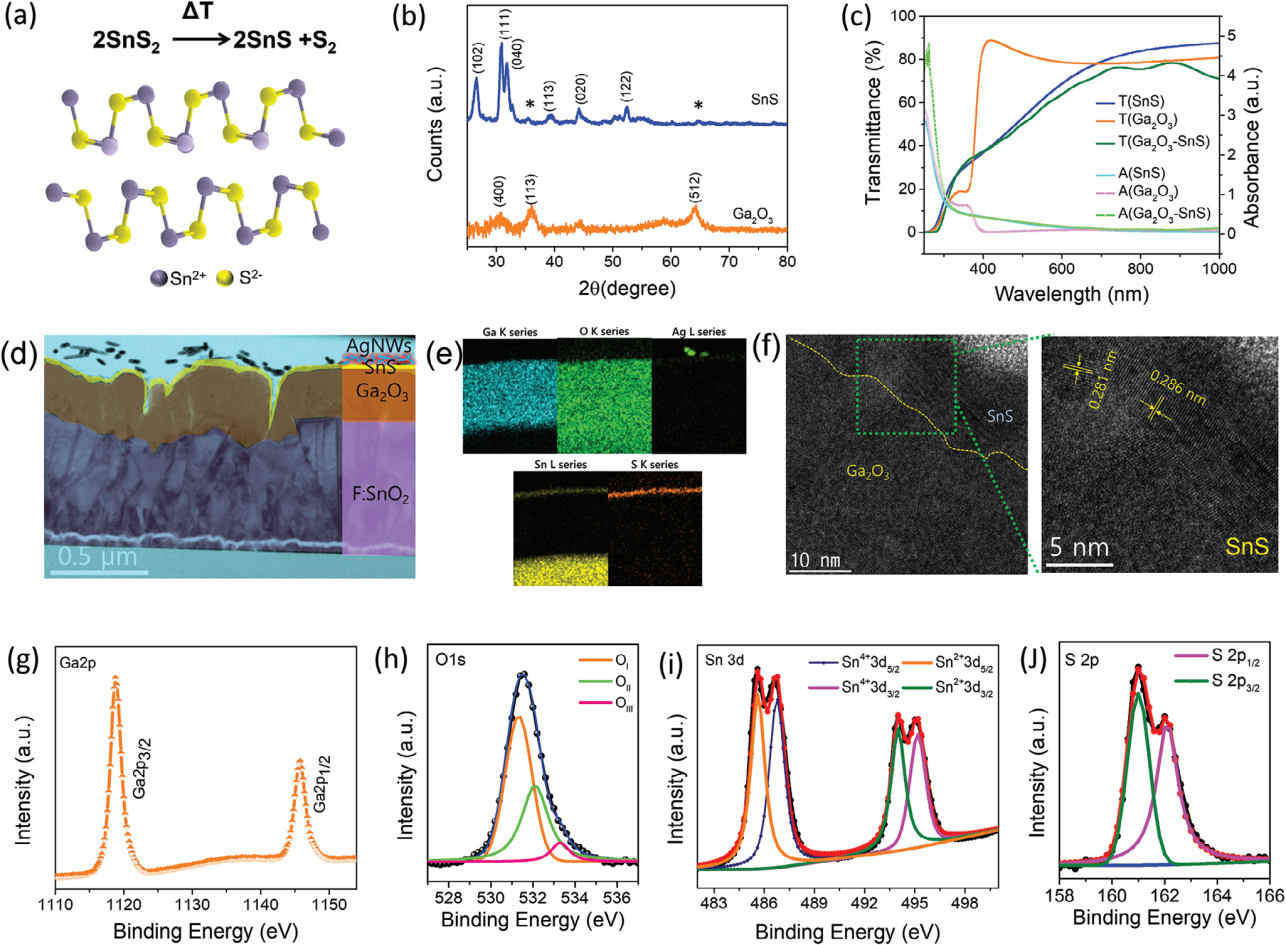
a) Structural transition of SnS_2_ phase to SnS phase under the PVD technique at 300 °C deposition temperature. b) XRD spectra of the Ga_2_O_3_ and SnS film grown on the glass substrate. c) Optical characteristics (transmittance and absorbance) of the SnS, Ga_2_O_3_, and Ga_2_O_3_/SnS heterojunction. d) High‐resolution TEM cross‐sectional image of the Ga_2_O_3_/SnS TPV. e) Elemental phase mapping of combined and individual Ga, O, Sn, S, and Ag elements obtained at the cross‐section. f) The interfacial TEM image depicts the interface formation between Ga_2_O_3_ and SnS. Zoomed‐in TEM micrograph of the SnS. XPS spectra of Ga_2_O_3_ film for g) Ga2P and h) O1s core level. XPS spectra of SnS film for g) Sn3d and h) S2p core level.

Tin disulfide (SnS_2_), a member of the IV–VI A group, is an n‐type layered semiconductor, and its structural transition to a p‐type 2‐D SnS semiconductor opens new research directions. The orthorhombic vdW SnS semiconductor was prepared by radio frequency sputtering at 300 °C deposition temperature. This is a single‐step process to obtain an orthorhombic SnS layer avoiding the afterward sulfurization like in chemical vapor deposition methods.^[^ ^31^ ^]^ The illustration of the structural transition of SnS_2_ to SnS is given in **Figure** **1**a with the formation of the SnS structure. The sputtered‐grown SnS_2_ particles dissociate into SnS and S layers and undergo a structural change at heating conditions.^[^ ^27^ ^]^ The dissociated sulfur may participate in maintaining the stoichiometry of SnS. Moreover, the excess S was also vented out from the system with the Ar flow after the deposition to prevent the SnS layered film with sulfur reaction. The single‐step SnS is deposited on the thick Ga_2_O_3_ film to achieve the transparent photovoltaic heterojunction, which acts as a transparent photovoltaic (TPV) device.

X‐ray diffraction (XRD) was performed over SnS and Ga_2_O_3_ to confirm the structural phases_,_ as shown in Figure 1b. The XRD peaks of (102), (111), (040), (113), (020), and (122) match well with JCPDS‐39‐0354 and previous reports confirming the orthorhombic structure of SnS of Pbnm(62) space group.^[^ ^27^, ^32^ ^]^ A slight signature peak of SnS_2_ identified with an asterisk (*) is also seen in the XRD spectrum of SnS film. The XRD peaks of (400), (113), (603), and (512) crystallographic planes were obtained, which verifies the β‐Ga_2_O_3_ monoclinic structure.^[^ ^33^, ^34^ ^]^ Raman spectroscopy was also performed to validate the SnS phase, and the Raman spectrum was recorded, as shown in Figure S1 (Supporting Information). The grown SnS film shows band peaks at 94, 174, and 226 cm^−1^, which concurs well with the reported spectra of SnS.^[^ ^27^ ^]^ A very slight peak at 312 cm^−1^ from the Raman spectrum indicates the SnS_2_ phase, which matches well with the XRD of SnS. Furthermore, UV–vis spectroscopy was performed to understand the optical properties of the SnS, Ga_2_O_3_, and the heterojunction of Ga_2_O_3_/SnS. Figure 1c shows the optical transmittance and absorbance of all three entities. Ga_2_O_3_ film has shown a high optical transmittance of ≈80% with a bandgap of 4.8 eV. In contrast, the ≈20 nm thin SnS film has shown lower optical transmittance and high absorption in the UV–Vis region. The heterojunction film (Ga_2_O_3_/SnS) shows a slight reduction in the optical transmittance; however, the absorption drastically increases in the UV region (<400 nm), exhibiting high photoresponse to the UV region. Figure 1d displays a cross‐sectional image of the TPV, which was captured using high‐resolution transmission electron microscopy (HRTEM). The fluorine‐doped tin oxide (FTO), Ga_2_O_3_, SnS, and AgNWs layers of the TPV can be clearly observed. Moreover, the inset in Figure 1d presents a schematic diagram of the device to provide a better understanding of the device cross‐section. Figure 1e depicts the elemental mapping of the device obtained through the energy dispersive spectrometer (EDS), which depicts the presence of Ga, O, Sn, S, and Ag elements in their respective films. It can be observed that a thin layer of SnS is deposited over a thick Ga_2_O_3_ layer, which provides the transparent nature of the TPV. Elemental mapping also ensures the uniform distribution of the elements in the device. Figure 1f shows an HRTEM image of the Ga_2_O_3_/SnS heterojunction, which depicts the heterojunction formation between Ga_2_O_3_ and SnS. The estimated *d*‐spacing of the SnS is calculated to be 0.286 and 0.281 nm, which corresponds to the (111) and (040) planes, confirming the orthorhombic SnS phase formation.^[^ ^35^, ^36^ ^]^ To gain insights into the material's stoichiometry and oxidation states, X‐ray photoelectron spectroscopy (XPS) has been performed over Ga_2_O_3_ and SnS films. Figure 1g,h shows the XPS spectra of Ga2p and O1s peak of Ga_2_O_3_. O1s peak of Ga_2_O_3_ was deconvoluted into O_I_ (531.2 ± 0.2 eV), O_II_ (532.2 ± 0.2 eV), O_III_ (533.2 ± 0.2 eV) peaks corresponding to the O^2−^ ions bonded to Ga^3+^ ions in Ga_2_O_3_ matrix, oxygen deficiency, and chemisorbed oxygen on the surface, respectively.^[^ ^37^ ^]^ Figure 1i shows the Sn3d core level peaks of the SnS. The Sn3d_5/2_ and Sn3d_3/2_ are deconvoluted into higher (4+) and lower (2+) oxidation states. The peaks centering at 485.6 and 494.2 eV are attributed to Sn3d_5/2_ and Sn3d_3/2_ states of SnS, respectively. The corresponding shoulder peaks centering at 486.8 and 495.2 eV are attributed to Sn3d_5/2_ and Sn3d_3/2_ states of SnS_2_, respectively.^[^ ^38^, ^39^ ^]^ Furthermore, the S2p peak of SnS is also deconvoluted, and peak centering at 161.0 and 162.2 eV can be attributed to the S2p_3/2_ and S2p_1/2_ states. XPS results agree with the XRD and Raman analysis which ensures the presence of Sn^4+^ in the SnS matrix.

### 2.2 Photovoltaic Analysis of TPV

**Figure 2:**
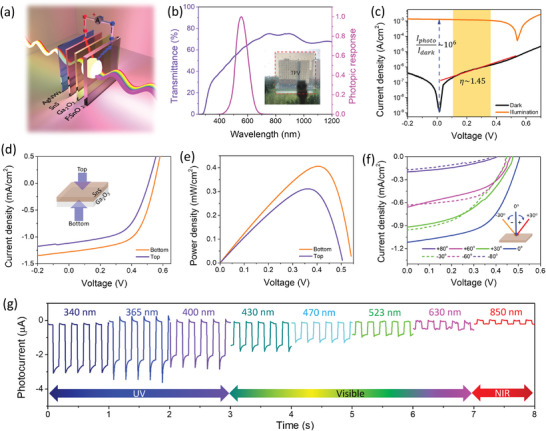
a) Schematic diagram of the TPV under light illumination. b) Transmittance and the photopic response of the complete TPV (AgNWs/SnS/Ga_2_O_3_/FTO). Inset: an image of the TPV. c) Semilogarithmic and linear I‐V curves under dark and illumination conditions. d) Linear J–V curve of the device demonstrating bifacial nature under illumination from bottom and top. e) Power density curve of the TPV device for the bottom and top illumination. f) Angle‐dependent linear J–V characteristics of the TPV device under UV light (365 nm) illumination with an intensity of 10 mW cm^−2^. g) Transient photocurrent response of the TPV under illumination from the UV (340 nm) to NIR (850 nm) exhibiting broadband photoresponse.

**Figure** **2**a presents the schematic diagram of the Ga_2_O_3_/SnS device with bifacial operation. The intrinsic n‐Ga_2_O_3_ and the p‐SnS form a heterojunction by the formation of space charge region enabling photo charge generation and their extraction for power and signals. Due to the transparent framework, this device possesses a see‐through device feature and can be confirmed by the transmittance spectra of the device, as shown in Figure 2b. The complete device (AgNWs/SnS/Ga_2_O_3_/FTO/glass) exhibited an average visible transmittance (AVT) of 56%, suitable for its integrated applications. The AVT assesses the transmission spectrum against the photopic response of the human eye and can be calculated as follows:

(1) $$\mathrm{AVT}\;=\frac{\int_{380\;\mathrm{nm}}^{780\;\mathrm{nm}}T\left(\lambda \right)P\left(\lambda \right)S\left(\lambda \right)d\lambda }{\int_{380\;\mathrm{nm}}^{780\;\mathrm{nm}}P\left(\lambda \right)S\left(\lambda \right)d\lambda }\;$$

where *λ* denotes the wavelength, *T* denotes the optical transmittance, *P* denotes the photopic response of the human eye, and *S* denotes the solar photon flux (AM 1.5G) (Figure 2b).

Furthermore, J–V measurement was performed on the proposed device in the dark and under different illumination conditions to demonstrate the photovoltaic characteristic of the Ga_2_O_3_/SnS‐based p‐n heterojunction TPV. The device was illuminated under a UV light source with a wavelength of 365 nm and light intensity of 10 mW cm^−2^. Junction formation can be confirmed by rectifying nature, as shown in Figure 2c. This result confirmed Ga_2_O_3_/SnS heterojunction with a diode ideality factor of 1.45, calculated in the range highlighted by the yellow region in Figure 2c and a dark saturation current of 1.45 nA. This results in a high photo‐to‐dark current ratio of 10^6^, suitable for photovoltaic receivers.

Figure 2d illustrates the linear J–V curve of the proposed device from the bottom and top illumination, demonstrating its bifacial power generation behavior. It exhibits a significantly high short‐circuit current density (Jsc) of 1.28 mA cm^−2^ and open circuit voltage (Voc) of 545 mV with a remarkably high fill factor (FF) of 58% with a skinny SnS layer under the illumination of UV light with the intensity of 10 mW cm^−2^ from the bottom. Upon illumination from the top side, the device records Jsc, Voc, and FF of 1.12 mA cm^−2^, 507 mV, and 55%, respectively. These results demonstrate the superiority of the proposed device over conventional SnS‐based photovoltaic devices, which typically exhibit a Voc deficit and poor FF.^[^ ^40^, ^41^, ^42^ ^]^ The output power of the proposed device is quantified based on the characteristic parameters of power conversion efficiency (PCE) and incident photon to current conversion efficiency (IPCE) of the photovoltaic receiver, which is calculated to be 4% and 44%, respectively, from the bottom illumination and 3.1% and 37% from the top illumination. Figure 2e shows the plot of the power output of the device. The maximum power output of the device is observed to be 0.41 and 0.31 mW cm^−2^ upon illumination from the bottom and top side, respectively, sufficient to power the IoT applications.^[^ ^43^ ^]^ The high PCE and power output of the device from the bottom and top illumination make it suitable for photovoltaic optoelectronic applications. Furthermore, the device was tested for the angular dependency of light sources on power production. The light illumination was performed from the top side, considering the top vertical view is 0^o^ illumination condition. Conventionally, the light source was rotated to the left (−) and to the right (+) at different angles, as shown in the inset of Figure 2f. The light source was moved from −80^o^ to +80^o^ and the J‐V curve was recorded by the device. The device has demonstrated a decrease in the J–V curve with the increased angle to both sides. The device has shown an excellent Jsc (0.20, 0.17 mA cm^−2^) and Voc(401, 374 mV) even at the illumination angle of ±80^o^. Conclusively, the device has shown outstanding angular photoresponse and power production characteristics. Figure 2g shows the transient photoresponse of the device under the illumination of a light source from UV (340 nm) to NIR (850 nm) region illustrating the broadband photo response of the device. The photodetector's performance parameters, such as spectral photoresponsivity (R), specific detectivity (D), linear dynamic range (LDR), rise and fall time, linear fitting parameter (α), and noise power spectral density (NEP) are calculated and shown in supporting information (Figure S2, Supporting Information). The device has demonstrated the highest R and D of 233 mA W^−1^ and 2.85 × 10^12^ Jones under the illumination of 365 nm wavelength radiation, respectively. Both R and D decreased with the increase in the illumination wavelength. The decaying behavior can be attributed to the interaction of low‐energy photons with the wide bandgap material (Ga_2_O_3_), resulting in poor photoresponse. The device showed the highest LDR and the fastest rise and decay time of 44.4 dB, 5.63, and 7.18 ms, respectively. Furthermore, α is calculated to provide the information of Ga_2_O_3_ and SnS interface, and it is calculated by considering power law (I∝P^α^). The α value was calculated to be 0.97, which is very close to one, indicating the reduced recombination centers at the interfaces and in the materials.^[^ ^44^, ^45^ ^]^ The lowest NEP was obtained under 365 nm illumination owing to its low dark current and high responsivity, and noise spectral density with respect to the frequency is also calculated and shown in Figure S2g (Supporting Information). These calculated photodetector performance parameters make the device appropriate for photodetection.

### 2.3 Omnidirectional Behavior of TPV

**Figure 3:**
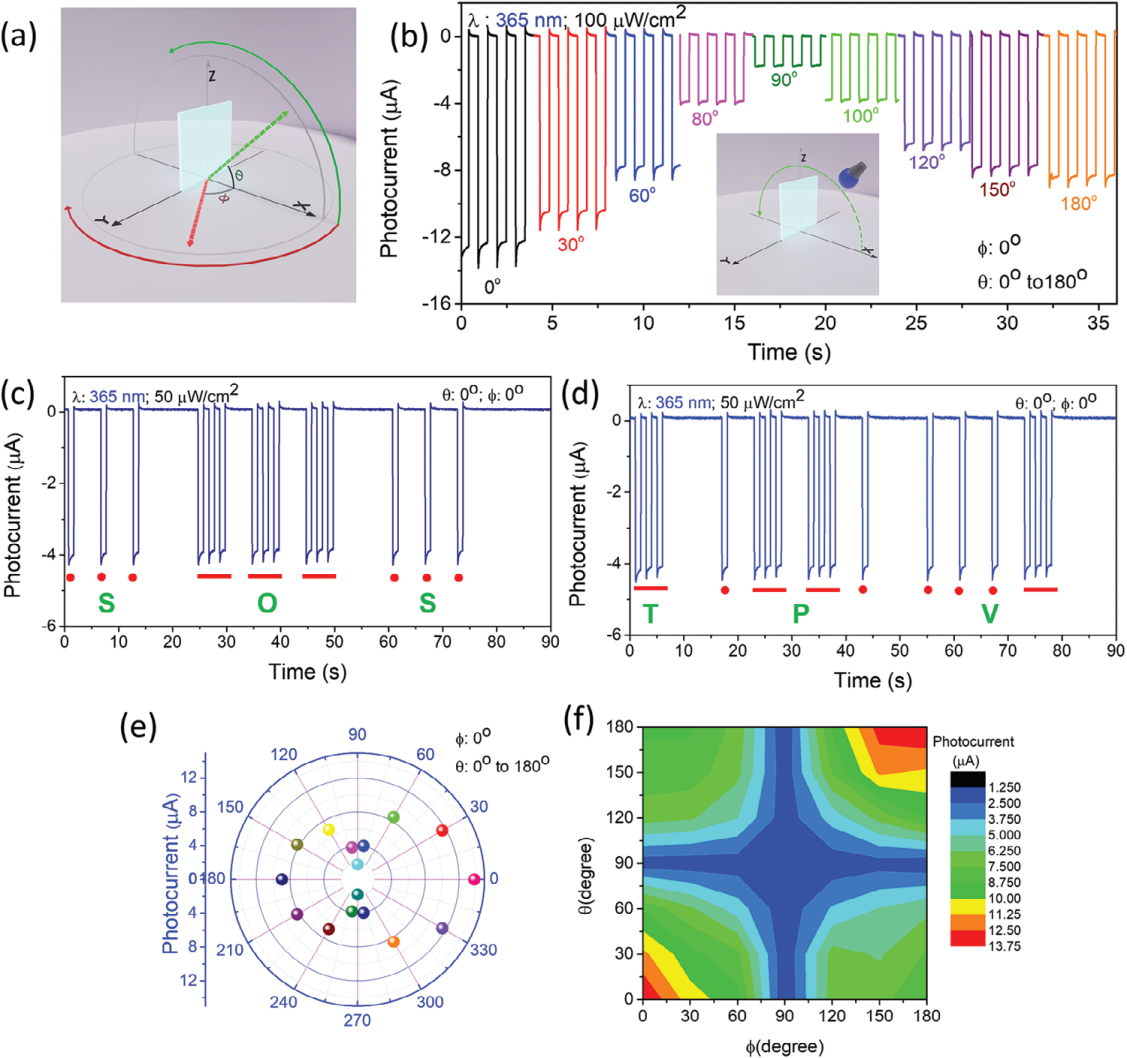
a) Polar coordinates represent the movement of the light source along the X, Y, and Z axes. b) Photocurrent response at ɸ = 0^o^ with the variation of θ from 0 to 180^o^. Morse code photoresponse deciphering c) SOS and d) TPV with a light source at θ= 0^o^ and ɸ = 0^o^. e) Photocurrent response of the device under the movement of the light source at ɸ = 0^o^ with the variation of θ from 0 to 360^o^. f) Contour plot of the device for photocurrent under the variation of θ and ɸ from 0^o^ to 180^o^.

On successfully demonstrating the photovoltaic behavior of the proposed device with high transparency, the TPV was subjected to omnidirectional photoresponse under the illumination of a light source ranging from visible to near‐infrared (NIR) wavelengths. Omnidirectional photoresponse is essential for developing a transparent receiving antenna or camera. The FoV refers to the maximum area of a sample that a camera can image; it depends on the focal length of the lens and sensor size. For a fixed focal length, a larger sensor presents a large FoV. Meanwhile, wFoV is essential for high‐quality imaging and for cameras that can capture a wide panoramic view. The FoV of the human eye ranges from ≈60^o^ to 155^o^.^[^ ^46^ ^]^ To capture the entire subject view, the subject angle must be less than the FoV of the camera.^[^ ^47^ ^]^ Lenses of suitable focal lengths and sensors are used to obtain a wFoV. However, such approaches increase the complexity of the design and make the system bulky. Such problems can be overcome by implementing an omnidirectional photodetector/sensor with a 360° FoV. The TPV is illuminated by light sources with different wavelengths (365, 523, and 850 nm), which are varied by varying the polar coordinates (r, ɸ, θ) between the light source and TPV to demonstrate 360^o^ wFoV under broadband light illumination. **Figure** **3**a depicts the movement of the light source in the X, Y, and Z directions with the variation angle, ɸ and θ, respectively. The distance, r, remains unchanged for the all‐angle variation to demonstrate omnidirectional photo sensing. Initially, ɸ was kept at 0^o^_,_ i.e., the normal of the device and light source was in the same direction and θ was varied from 0^o^ to 180^o^ (Figure 3b). The photocurrent response of the device was recorded at different angles under the illumination of the light source with a wavelength of 365 nm and intensity of 100 µW cm^−2^, as shown in Figure 3b. Remarkably, the device recorded a signal even at (ɸ=0^o^, θ=90^o^). Thus, it can be observed that the device can record signals even when light falls perpendicularly on the glass side. The photocurrent response was observed to continuously decrease from 0^o^ to 90^o^ and then constantly increase from 90^o^ to 180^o^, exhibiting maximum photo sensing along the line of sight. Similarly, the photocurrent response of the TPV was also recorded at θ=0^o^, and ɸ was varied from 0^o^ to 180^o^. Figure S3a (Supporting Information) presents the schematic diagram of the movement of the light source for θ =0^o^ and ɸ variation from 0^o^ to 180^o^. The ɸ variation photoresponse behavior is identical to that of the θ variation. The photocurrent decreases from 0^o^ to 90^o^ and increases from 90^o^ to 180^o^, as shown in Figure S3b (Supporting Information). The device also recorded a significant photo signal even at (ɸ=90^o^, θ=0^o^). Morse code of SOS and TPV was used as a transmitted encoded signal to demonstrate the PC. The Morse code was sent in the form of light pulses, which the TPV collectively received, and the photocurrent was recorded for the SOS and TPV signals, as shown in Figure 3c,d, respectively. The projected light intensity on the device was 50 µW cm^−2^, which is considerably low, making the device ultra‐sensitive to the UV regime. The SOS and TPV Morse code signals were recorded for (ɸ, θ) at (0^o^, 180^o^), (0^o^, 90^o^), (90^o^, 0^o^), and (90^o^, 180^o^), and are presented in Figure S4 (Supporting Information). Evidently, Figure 3 and Figure S4 (Supporting Information) clearly demonstrate the omnidirectional receiver behavior, which generates photosignals under light illumination at various values of (ɸ, θ). The visible and NIR light sources with wavelengths of 523 and 850 nm, respectively, were used to demonstrate the TPV broadband wavelength omnidirectional light response. Figures S5 and S6 (Supporting Information) depict the photoresponse and Morse code embedded SOS and TPV signal detection under the illumination of the light sources with wavelengths of 523 and 850 nm, respectively, for different values of (ɸ, θ). The TPV exhibits a remarkable ability to generate photosignals under broadband wavelength light illumination, making the device applicable for broadband light PCs. A better depiction of the device response to collect Morse code of TPV for various angles of incident photons of different wavelengths is shown in Figure S7 (Supporting Information).

A polar contour plot was drawn for the photocurrent for different values of (ɸ, θ) to demonstrate the omnidirectionality of the TPV, as shown in Figure 3e. The omnidirectionality of the photosensing is demonstrated under the movement of the light source at ɸ = 0^o^ while varying θ from 0^o^ to 360^o^ (Figure 3e), and at θ = 0^o^ while varying ɸ from 0^o^ to 360^o^ (Figure S8a, Supporting Information). The light source positioned at (ɸ=0^o^, θ=90^o^) and (ɸ=90^o^, θ=0^o^), which is perpendicular to the device from the top side or from the left or right side, is called a blind spot, as shown in Figure S8 (Supporting Information). At the blind spot, the device is expected to have no signal due to the low exposure of the film to the light source. However, the developed TPV has recorded photo signals even at this blind spot. The linear contour plot is plotted to understand the omnidirectionality corresponding to the photocurrent and responsivity (Figure 5c; Figure S8b, Supporting Information). The photocurrent of the device was recorded at all possible angle combinations by varying (ɸ, θ), and a matrix was created for the linear contour plot of the photocurrent. The linear contour plot is drawn with the photocurrent contour plot due to the correlation between photocurrent and responsivity. The TPV exhibits the highest responsivity of 138 mA W^−1^ at a normal incidence of (ɸ=0^o^, θ=0^o^) to the device and the lowest responsivity of 17 mA W^−1^ at the blind spot (ɸ=90^o^, θ=90^o^) of the device (Figure S9, Supporting Information). The TPV recorded the responsivity at all polar coordinates, and no blind spot was observed for the proposed device. This high omnidirectionality and wFoV make the TPV an essential component in photocommunication and building integrated photovoltaic systems.

Existing photodetectors used for PC exhibit limited spectral photoresponse and omnidirectionality. Liu. et al. developed a CsPbBr_3_ perovskite photodetector for visible light communication with the highest responsivity of 110 mA W^−1^, with limited omnidirectionality.^[^ ^48^ ^]^ Similarly, Bao et al. developed a halide‐perovskite‐based photodetector for optical communication and demonstrated data transfer based on binary signals. However, it exhibited low omnidirectionality, and the spectral response was limited to 460 nm.^[^ ^49^ ^]^ Conversely, the proposed device demonstrates highly omnidirectional photosensing under broadband light source illumination with the highest responsivity at normal incidence to the device to lowest responsivity at the blind spot of the device at self‐biased conditions while presenting the highest AVT of 56%. Unlike previous studies,^[^ ^23^, ^50^ ^]^ the fabrication process did not require nanostructure formation or nanostructure patterning to obtain omnidirectionality.

### 2.4 Charge Transport Physics Analysis

Impedance spectroscopy of emerging photovoltaics provides signature information for emerging devices, including built‐in potential, number of capacitive regions, diffusion components, and shunt resistance.^[^ ^51^, ^52^, ^53^ ^]^ This method has been applied to Ga_2_O_3_/SnS TPV devices by measuring impedance spectra for the dark and various photon fluxes. Evident from the XPS analysis, Ga_2_O_3_ and SnS both film possess photoactive defect states (Sn, Ga, and O vacancies); their role in the junction modulation can be revealed by steady‐state illumination.

**Figure 4:**
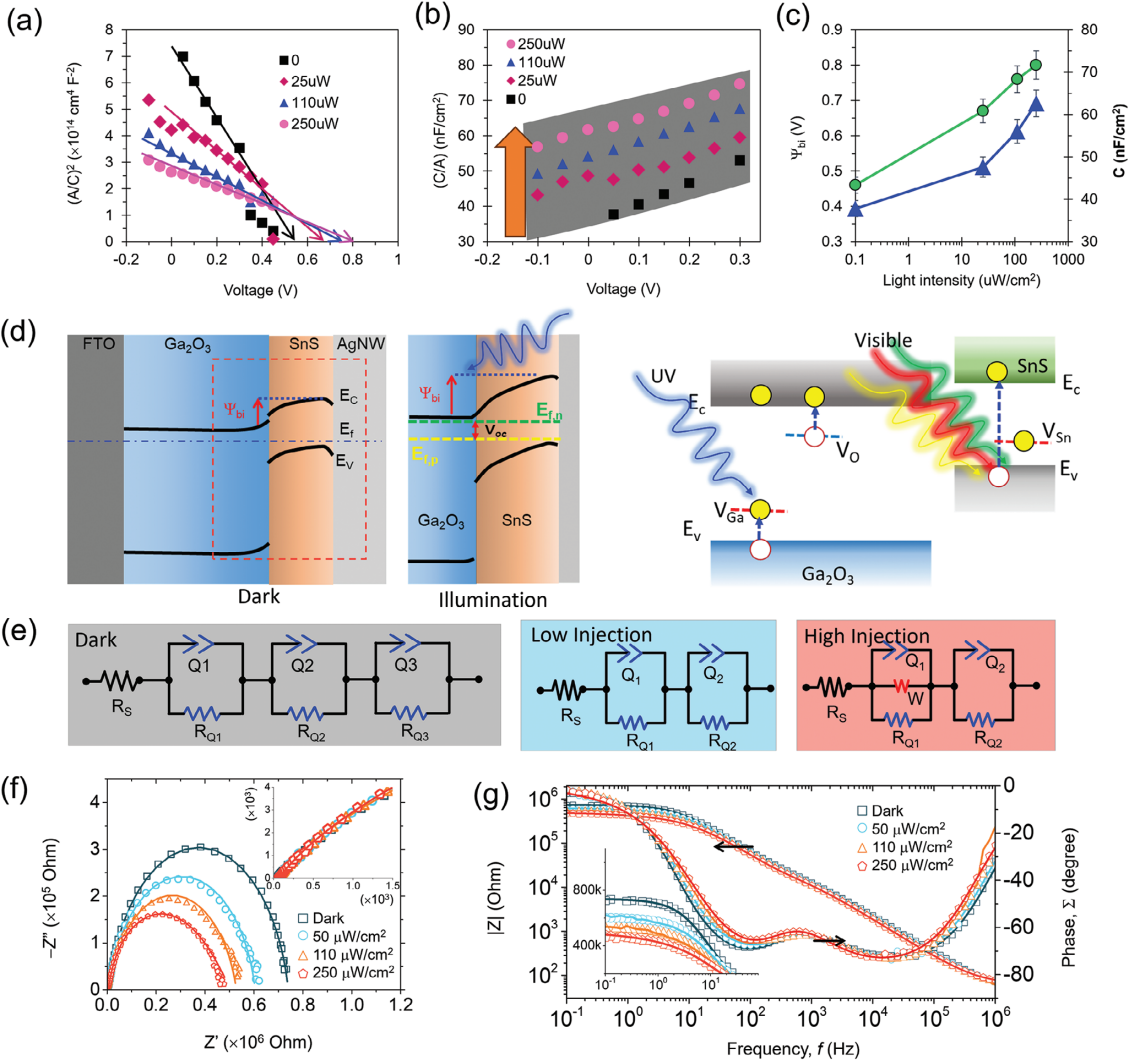
a) Mott–Schottky (1/C^2^‐V) curve and b) aerial capacitance versus voltage characteristics of the device under dark and different illumination conditions. c) Flat band and capacitance behavior to the illumination photon flux. d) Energy band alignments of different interfaces (FTO‐Ga_2_O_3_, Ga_2_O_3_‐SnS, SnS‐AgNW) under dark and illumination conditions. Photon‐induced charge injection under illumination. e) Equivalent circuits for dark, low‐injection, and high‐injection conditions. f) Cole–Cole plot and g) Bode amplitude and bode phase plot under dark and illumination conditions.

Mott–Schottky characteristics of the diode device confirm the built‐in potential (Ψ_bi_) by the (A/C)^2^ versus voltage plot, as shown in **Figure** **4**a. This result shows significant modulation of capacitive behavior and improved Ψ_bi_. As visible, the flat‐band potential (V_FB_) value of 0.53 V for the dark improved to 0.8 V by illumination of 250 µW/cm^‐2^. This V_FB_ value can confirm the role of photo‐excited defects in enhancing the Ψ_bi_. On the other hand, the aerial capacitance of the device significantly increased, from 35 to 65 nF, nearly doubled of the device under the illumination for the low bias conditions, as shown in Figure 4b. The role of light illumination on the built‐in potential and device capacitance is shown in Figure 4c. This result shows the logarithmic nature of Ψ_bi_ modulation for photon flux density. Figure 4d illustrates the energy band‐edges of the FTO/Ga_2_O_3_/SnS/AgNW device for the dark and illumination conditions. It depicts the increased Ψ_bi_ and band‐edge redistribution by the illuminations. It shows the photon‐assisted defect carrier injection where Ga, O, and Sn vacancies are crucial.^[^ ^54^ ^]^ The significant change in Ψ_bi_ and aerial capacitance can provide evidence of photon‐assisted defect injection. The bandgap and equivalent circuit analysis using impedance spectroscopy revealed the photo capacitance resulting from the various interfacial circuits of the nanoplatelets of SnS and SnS/Ga_2_O_3_ and SnS/AgNWs interface. Equivalent circuits shown in Figure 4e were employed for the dark, low injection, and high injection conditions to fit this complex nature of Cole–Cole plots. Figure 4f shows the cole–cole plot of the Ga_2_O_3_/SnS device under dark and various light intensities. An inset in Figure 4f shows how well the fitting is performed for dak and illumination conditions. A significant reduction in the shunt resistance from 0.73 to 0.49 MΩ is seen from dark to illumination conditions attributing to the significant change in the photoinduced injection of defect carriers in the device. All fitted parameters are shown in Table S1 (Supporting Information). Figure 4g depicts a Bode amplitude and phase plots recorded for the Ga_2_O_3_/SnS device to identify the nature of inhomogeneities associated with defects, grain boundaries, diffusion, etc. The Bode amplitude decreases by nearly half in the low‐frequency region, which is a capacitive‐dominated region from dark to illumination conditions. It elucidates the significant injection photoactivated defects. The low‐frequency maxima and high‐frequency maxima in the Bode phase plot originate from the interfaces and diffusion around the electrodes and from grains. Figure 4f clearly exhibits lower and higher frequency maxima originating from the contribution of interfaces of SnS and Ga_2_O_3_ and the nanoplates structure of SnS, which form grain boundaries. However, for lower frequency maxima, the peak shifts to the lower side with going from low to high injection attributing to the decrease in carrier lifetime.

### 2.5 Visual‐Speech Communication System

**Figure 5:**
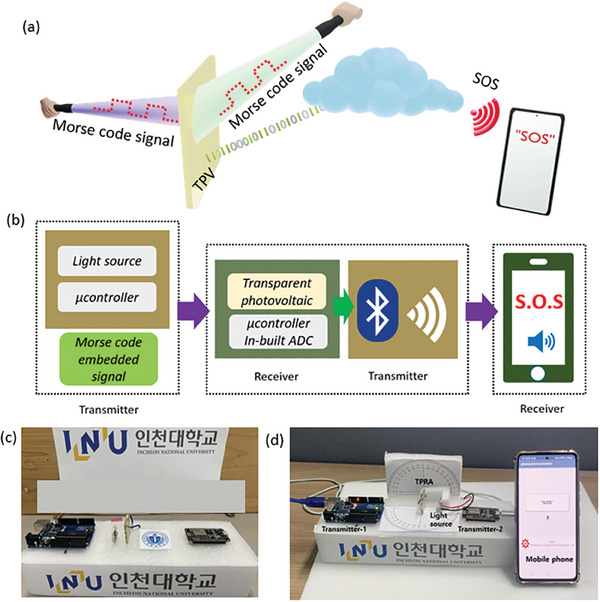
a) Depiction of the concept behind the proposed device. b) Schematic diagram of the module developed for visual and speech PC, c) Visual‐speech PC module. d) Visual‐speech PC module to record the omnidirectional behavior of the TPV.

A large proportion of the global population faces visual and auditory impairments. Deafness and hearing loss are the most widespread. More than 1.5 billion people, 20% of the world's population, have auditory impairments. The number could rise to 2.5 billion, and one in four people may have auditory problems by 2050.^[^ ^55^ ^]^ In addition to auditory impairments, more than 2.2 billion people globally have a visual impairment, and nearly one billion people have a vision impairment that can be cured or prevented.^[^ ^56^ ^]^ Therefore, we used the TPV to receive photo signals from all directions and transmit them for visual and speech generation in electronic devices, such as mobiles or computer displays, demonstrating its practical application in visual and speech communication. This feature can be implemented in assistive devices used by people with visual and auditory impairments. The high transparency of the TPV makes it suitable to embed on the windows and walls of buildings for energy production and omnidirectional photosensitivity. The TPV demonstrates onsite power generation and omnidirectional photosensing behavior. Moreover, a module has been developed to demonstrate its practical application in daily tasks. **Figure** **5**a presents an animated representation of the fundamental concept behind the TPV. The high transparency of the device makes it suitable to be installed on the walls or windows of a building to generate onsite power under continuous illumination. Furthermore, it can also serve as an omnidirectional transparent receiver antenna. Figure 5b presents the schematic diagram of the module developed to demonstrate visual and speech communication. Figure 5c illustrates the developed visual and speech communication module. Furthermore, the module is modified with a moveable light source arrangement to demonstrate omnidirectional visual and speech communication, as shown in Figure 5d. The video demonstration of the visual and speech communication for words such as SOS and TPV is presented in the supporting video for different ɸ and θ values.

## 3 Conclusion

This study provides valuable insight into the development of an omnidirectional transparent photovoltaic device. The proposed TPV demonstrates a high average visible transmittance of 56%, which makes it suitable for bifacial onsite energy production. The TPV overcomes the limited FoV of the existing photoelectric devices and achieves 360° FoV owing to the feature of omnidirectionality. The device has shown an ultra‐sensitivity to the UV region attributed to the mechanism of the photon‐injected defect. The presence and role of defects were studied by XPS and impedance spectroscopy. The developed TPV can be used in various types of PC applications, such as speech devices for visually impaired people, display devices for hearing impaired people, or safety window displays. Future studies will focus on equipping the receiver with a self‐powered support mode.

## 4 Experimental Section

### Device Fabrication

To fabricate the Ga_2_O_3_/SnS‐based TPVs, an F:SnO_2_‐coated glass (Sigma‐Aldrich‐735159) was used as a substrate after sequential cleaning with acetone, methanol, and distilled water. A high‐purity Ga_2_O_3_ (99.99%, iTASCO) target was used to grow the β‐Ga_2_O_3_ film. The Ga_2_O_3_ was deposited via a radio frequency (RF) sputtering system (Solarlight Ltd, Korea) at a deposition power of 200 W under an operating pressure of 5 mTorr for one hour at room temperature. The room‐temperature grown Ga_2_O_3_ was subjected to rapid thermal annealing at 500 °C for 10 min in an Ar ambient environment to obtain β‐Ga_2_O_3_. Subsequently, a SnS film with a ≈15 ± 2 nm thickness was deposited on the room‐temperature grown Ga_2_O_3_. A target composed of SnS_2_ particles (SnS_2_, 99.999%, iTASCO) was used to obtain a p‐type SnS film. The SnS films were deposited via RF magnetron sputtering at 50 W under an operating pressure of 6 mTorr at 300 °C. The Ar flow was maintained at 50 sccm to maintain the operating pressure of 5 mTorr for Ga_2_O_3_ and 6 mTorr for SnS. To deposit ≈15 nm SnS thin films, the deposition time was maintained at 150 s. On depositing the SnS film, the substrate was cooled to room temperature under an Ar flow of 50 sccm while maintaining a pressure of 6 mTorr in the chamber. The top electrode was composed of silver nanowires (AgNWs). AgNWs with a diameter of ≈20 nm and length of ≈20 µm (Nanopyrix) were spin‐coated over the Ga_2_O_3_/SnS heterojunction and then dried, providing a fully transparent device.

### Characterization

HRTEM was used to capture the cross‐sectional view of the Ga_2_O_3_/SnS TPVs. The cross‐sectional HRTEM sample analysis was prepared via Ar ion beam milling (Quanta 3D FEG). A cross‐sectional image of the Ga_2_O_3_/SnS TPV was captured using TEM (JEOL 2100F) at an operating voltage of 200 kV. The elemental compositions were recorded using the EDS (OXFORD X‐MAX). Raman spectroscopy (JOBIN YVON/LabRAM Hr800) was performed with an excitation source of 514.5 nm to verify the SnS phase. XRD (SmartLab, Rigaku) was performed using Cu–Kα radiation (*λ* = 1.54059 Å) to verify the Ga_2_O_3_ phase composition. XPS (ULVAC‐PHI) was used to understand the material's stoichiometry and electronic binding states. The transmittance spectra of the Ga_2_O_3_/SnS TPVs were measured through UV–vis reflectance spectrophotometry (UV‐2600, Shimadzu). The current‐voltage measurements were recorded using a source measuring unit (SMU, Keithley 2400) and a potentiostat/galvanostat (PGStat, ZIVE SP2 WonA Tech.). A power meter (Mcscience‐K101) was used to measure the TPV performance.

### Visual and Speech PC

A handmade setup was prepared to demonstrate visual and speech PC, as shown in Figure S1 (Supporting Information). The UV (365 nm) light source was used as a transmitter with the Arduino. The Ga_2_O_3_/SnS transparent photovoltaic device was used as an omnidirectional TPV receiver, and another Arduino was used as a transmitter to transmit the decoded signal to the cell phone via Bluetooth or Wi‐Fi clouding.

## Conflict of Interest

The authors declare no conflicts of interest.

## Supporting information

Supporting Information

Supplemental Video 1

## Data Availability

The data that support the findings of this study are available from the corresponding author upon reasonable request.
