# Supplementary material for: Van Der Waals Semiconductor Based Omnidirectional Bifacial Transparent Photovoltaic for Visual‐Speech Photocommunication
Source: Adv Sci (Weinh). 2023 Dec 11;11(7):2306408. doi: 10.1002/advs.202306408 (PMC10870018; doi:10.1002/advs.202306408)
Supplement: Supplementary file 1 — Supporting Information [file ADVS-11-2306408-s001.pdf]

ADVANCED SCIENCE
Open Access

Supporting Information

for *Adv. Sci.*, DOI 10.1002/advs.202306408

Van Der Waals Semiconductor Based Omnidirectional Bifacial Transparent Photovoltaic for Visual-Speech Photocommunication

Naveen Kumar, Thanh Tai Nguyen, Junsik Lee, Malkeshkumar Patel, Priyanka Bhatnagar, Kibum Lee and Joondong Kim**

Supporting information

Van der Waals semiconductor based omnidirectional bifacial transparent photovoltaic for visual-speech photocommunication

Naveen Kumar[+], Thanh Tai Nguyen[+], Junsik Lee, Malkeshkumar Patel, Priyanka Bhatnagar, Kibum Lee and Joondong Kim**

Dr. N. Kumar, Dr. T. T. Nguyen, Mr. J. Lee, Dr. M. Patel, P. Bhatnagar, Prof. J. Kim

[1]Photoelectric and Energy Device Application Lab (PEDAL) and Multidisciplinary Core Institute for Future Energies (MCIFE),

[2]Department of Electrical Engineering, Incheon National University, Incheon 22012, Korea

Email: joonkim@incheon.ac.kr

Mr. K. Lee
Solarlight Ltd., 119 Academy Rd. Yeonsu, Incheon, 22012, Republic of Korea
Email: solarlight_1@naver.com (K. Lee)

[+]N. Kumar and T. T. Nguyen equally contributed to this work.

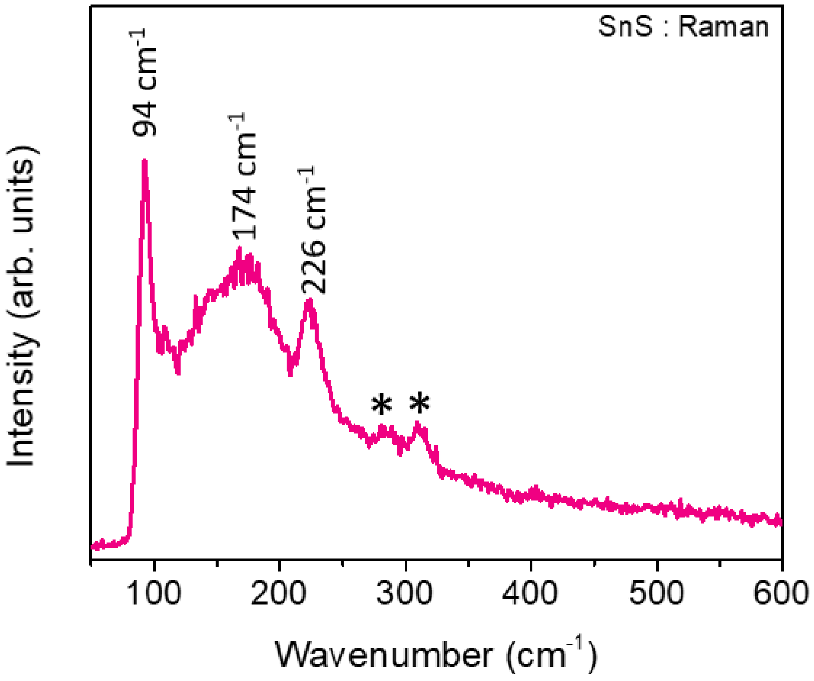

Figure S1. Raman spectrum of SnS film.

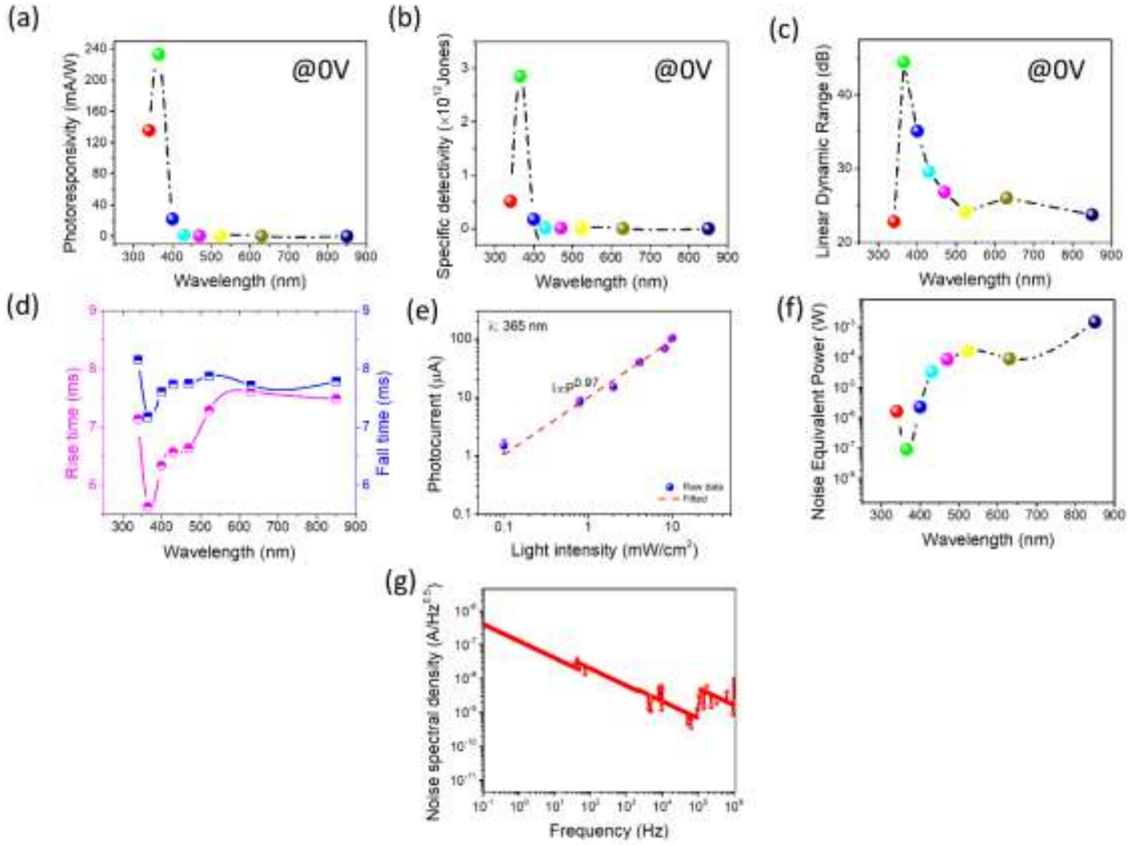

Figure S2: (a) Photoresponsivity, (b) specific detectivity, (d) linear dynamic range, (d) rise and fall time, (e) power law relation between photocurrent and illuminated light intensity, (f) noise equivalent power, and (g) noise spectral density calculated for the Ga_2O_3/SnS TPV.

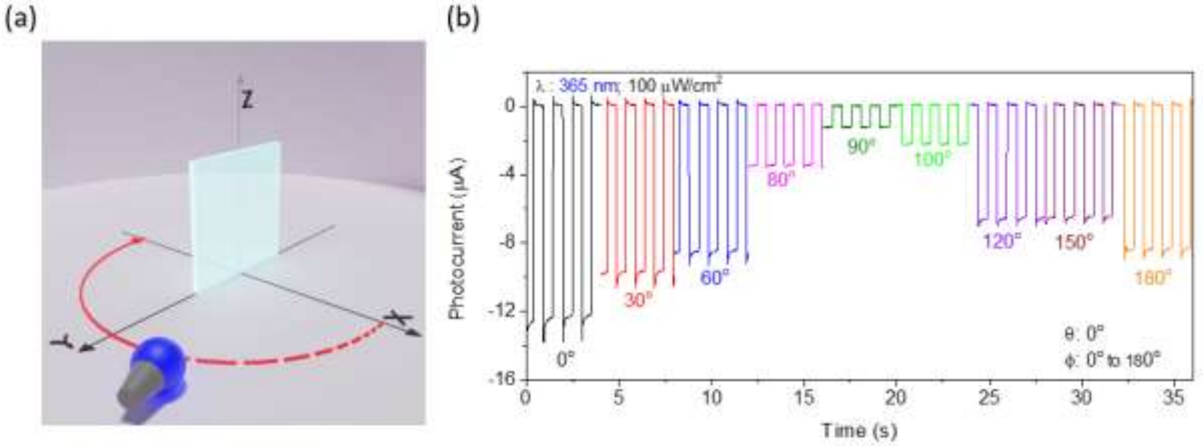

Figure S3. (a) Representation of movement of the light source and (e) photocurrent response at θ=0° with the variation of ϕ from 0 to 360°.

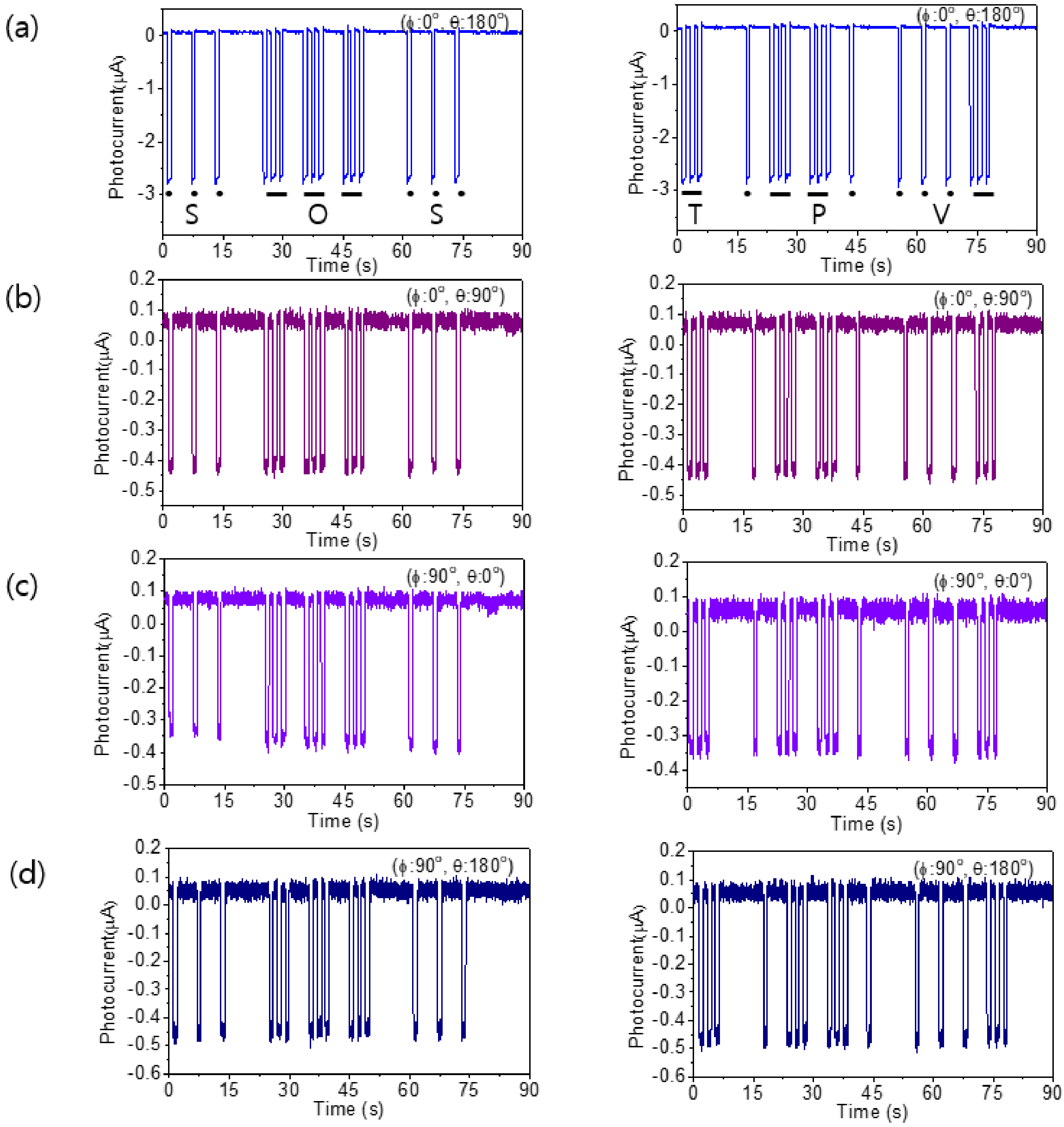

Figure S4. Morse code-embedded photosignal for SOS and TPV under UV light illumination at (a) (ϕ:0°, θ:180°), (b) (ϕ:0°, θ:90°), (c) (ϕ:90°, θ:0°), and (d) (ϕ:90°, θ:180°) recorded by TPV.

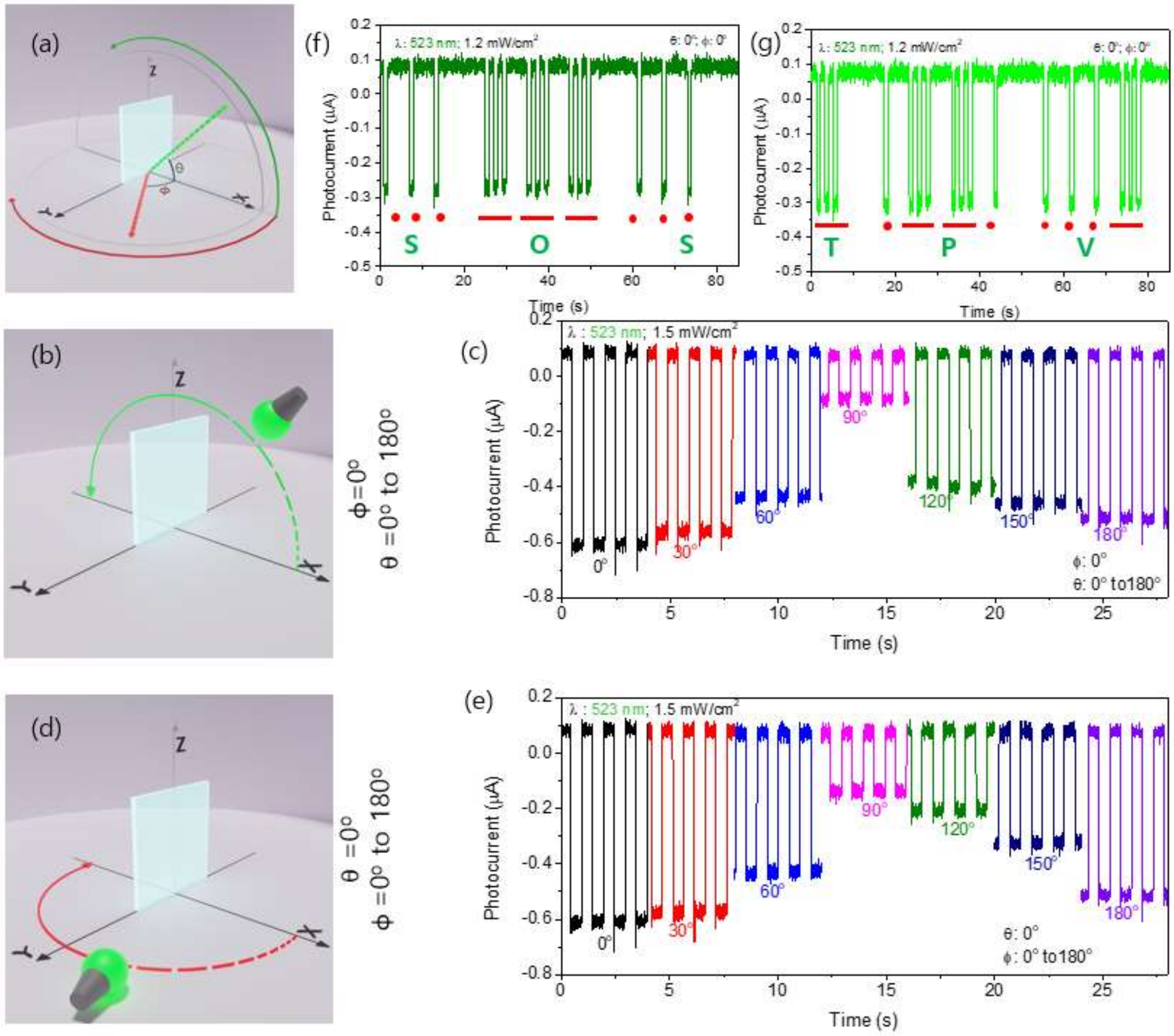

Figure S5. (a) Polar coordinates representation of the movement of the light source with wavelength of 523 nm along the X, Y, and Z axes. (b) Representation of light source movement and (c) photocurrent response at $\phi=0^o$ with variation of θ from 0 to 180°. (d) representation of movement of the light source and (e) photocurrent response at $\theta=0^o$ with variation of ϕ from 0 to 360°. Morse code photoresponse decoding of (f) SOS and (g) TPV with a light source positioned at $\theta=0^o$ and $\phi=0^o$.

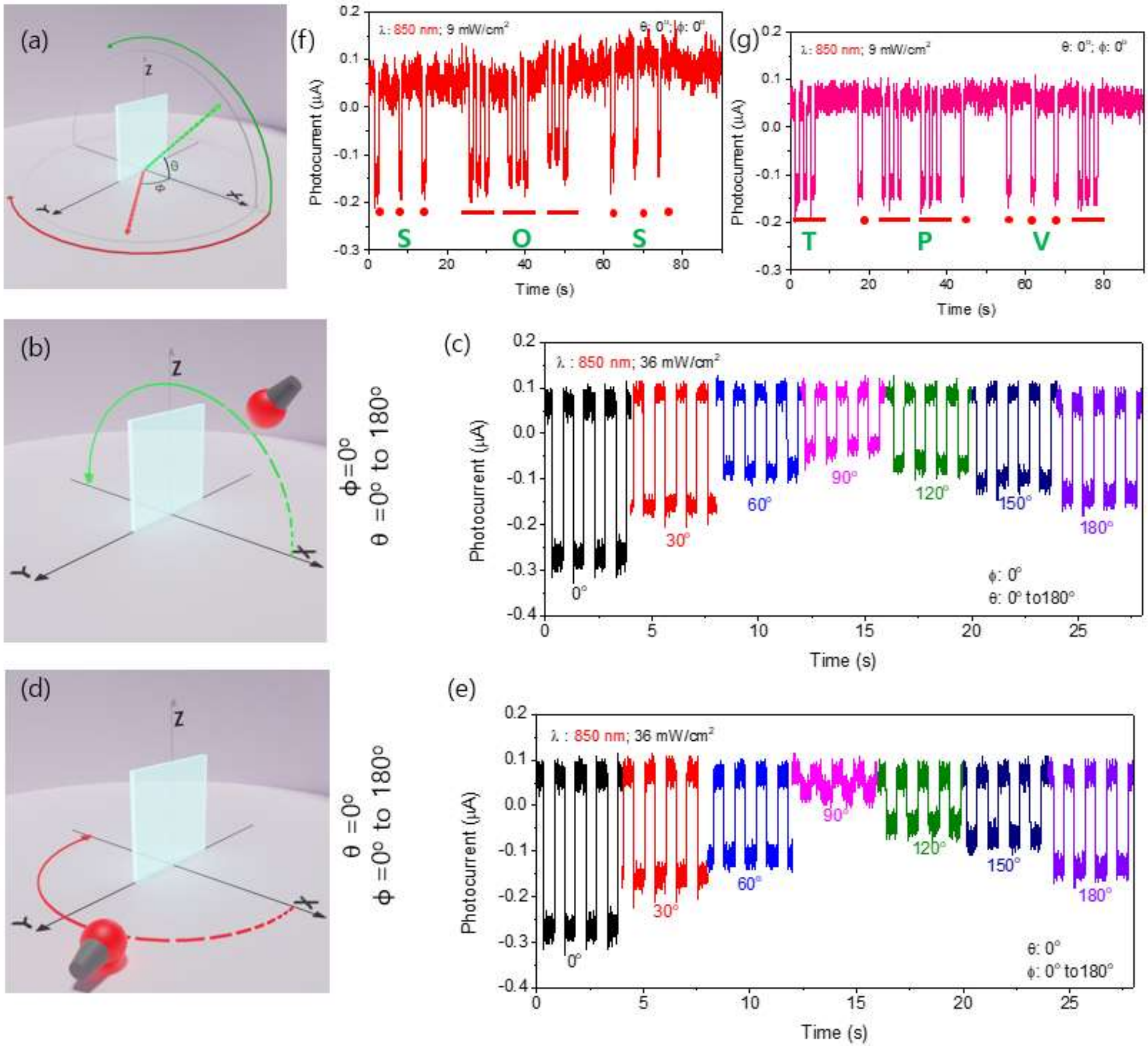

Figure S6. (a) Polar coordinates representation of the movement of the light source with wavelength of 850 nm along the X, Y, and Z axes. (b) Representation of movement of the light source and (c) photocurrent response at ϕ=0° with variation of θ from 0 to 180°. (d) Representation of movement of the light source and (e) photocurrent response at θ=0° with variation of ϕ from 0 to 360°. Morse code photoresponse decoding of (f) SOS and (g) TPV with a light source positioned at θ=0° and ϕ=0°.

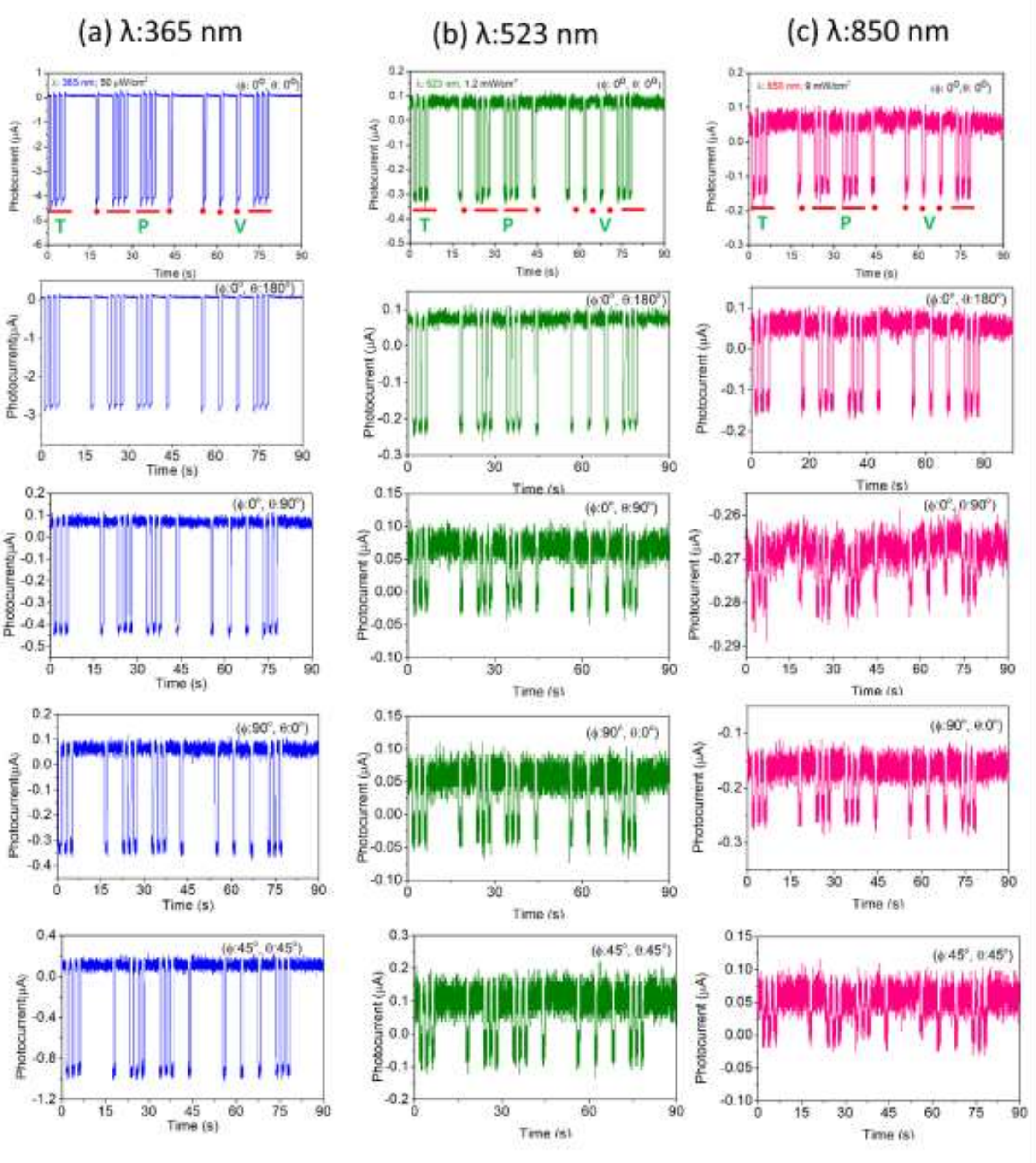

Figure S7. Morse code detection for various angles of incident photons such as (ϕ:0°, θ:0°), (ϕ:0°, θ:180°), (ϕ:0°, θ:90°), (ϕ:90°, θ:0°), and (ϕ:45°, θ:45°) under the illumination of light source of wavelength (a) 365, (b) 523, and (c) 850 nm.

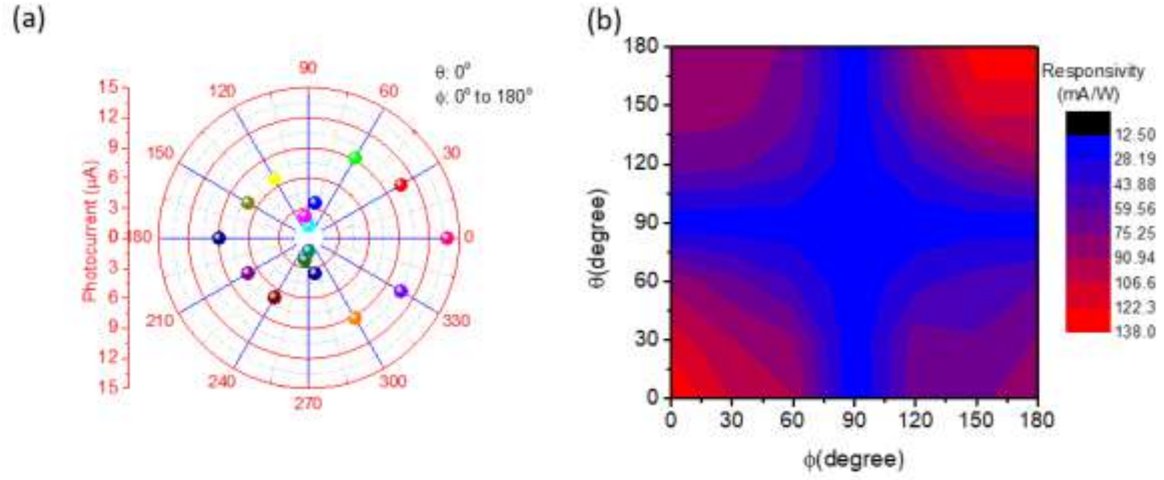

Figure S8. (a) Photocurrent response of the device under the movement of the light source at $\theta=0^{o}$ with variation of ϕ from 0 to 360°. (b) Contour plot of the device for responsivity under the variation of θ and ϕ from 0° to 180°.

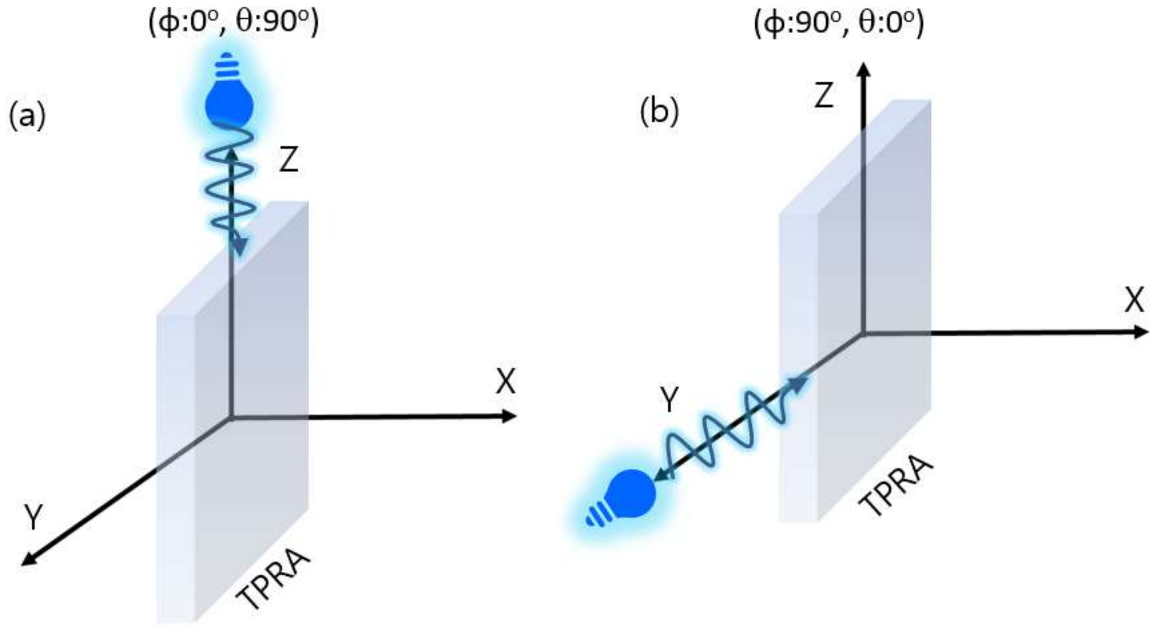

Figure S9. Polar depiction of light source arrangement with blind spots at the (a) (ϕ:0°, θ:90°) and (b) (ϕ:90°, θ: 0°) coordinates.

Table S1. Fitted parameters evalauted from the fitting of cole-cole plot under dark and illumination conditions from differetn fluxes.

**Parameters**	**Unit**	**Dark**	**25 µW**	**110 µW**	**250 µW**
**Rs**	Ω	54.5	54.7	55.3	62.3
**R1**	Ω	732683	6386.4	5349.5	495718.6
**Q1(γ)**	n Ω^{-1}	51.84 (0.882)	84.94 (0.850)		
**R2**		77041.16	598494.9	522770.0	10393.2
**Q2(γ)**	n Ω^{-1}	3.42 (0.809)	60.95 (0.866)	80.34 (0.836)	85.40 (0.823)
**R3**		7251.6			
**Q3(γ)**	n Ω^{-1}	72.53 (0.85)			
**C**	nF			17.44	25.51
**W**	µ Ω^{-1}			1.343	0.189

Change in the supporting information:

Calculations for R, D*, LDR, and NEP:

Photoresponsivity can be calculated by the equation 1[1]:

$$R(\lambda) = \frac{I_{photo} - I_{dark}}{P_{inc}A} \qquad [1]$$

Where I_{photo}, I_{dark}, P_{inc}, and A are the photocurrent, dark current, incident light intensity, and effective area, respectively.

When the dark current is dominated by the shot noise, specific detectivity can be calculated by equation 2 [1]:

$$D* = \frac{R(\lambda)}{\sqrt{2qI_{dark}}} \qquad [2]$$

Where, Where, $R(\lambda)$, I_{dark}, and q are photoresponsivity, dark current, and electron charge, respectively.

Linear dynamic range of a photodetector can be calculated as:

$$LDR = 20\log(\frac{I_{photo}}{I_{dark}}) \qquad [3]$$

NEP of a photodetector can be calculated using the equation [2]:

$$NEP = \frac{i_n}{R(\lambda)} \qquad [4]$$

Where i_n and $R(\lambda)$ are the dark current noise and photoresponsivity, respectively.

[1] C. H. Ji, K. T. Kim, S. Y. Oh, *RSC Adv.* **2018**, *8,* 8302.

[2] C. Bao, Z. Chen, Y. Fang, H. Wei, Y. Deng, X. Xiao, L. Li, J. Huang, *Adv. Mater.* **2017**, *29,* 1703209.
